# Estimating polymorphic growth curve sets with nonchronological data

**DOI:** 10.1002/ece3.6528

**Published:** 2020-07-20

**Authors:** Kai Moriguchi

**Affiliations:** ^1^ Faculty of Agriculture and Marine Science Kochi University Nankoku City Japan

**Keywords:** anamorphic, asymptote, growth model, maximum likelihood estimation, polymorphic, underdispersion

## Abstract

1. When we collect the growth curves of many individuals, orderly variation in the curves is often observed rather than a completely random mixture of various curves. Small individuals may exhibit similar growth curves, but the curves differ from those of large individuals, whereby the curves gradually vary from small to large individuals. It has been recognized that after standardization with the asymptotes, if all the growth curves are the same (anamorphic growth curve set), the growth curve sets can be estimated using nonchronological data; otherwise, that is, if the growth curves are not identical after standardization with the asymptotes (polymorphic growth curve set), this estimation is not feasible. However, because a given set of growth curves determines the variation in the observed data, it may be possible to estimate polymorphic growth curve sets using nonchronological data.
2. In this study, we developed an estimation method by deriving the likelihood function for polymorphic growth curve sets. The method involves simple maximum likelihood estimation. The weighted nonlinear regression and least‐squares method after the log‐transform of the anamorphic growth curve sets were included as special cases.
3. The growth curve sets of the height of cypress (*Chamaecyparis obtusa*) and larch (*Larix kaempferi*) trees were estimated. With the model selection process using the AIC and likelihood ratio test, the growth curve set for cypress was found to be polymorphic, whereas that for larch was found to be anamorphic. Improved fitting using the polymorphic model for cypress is due to resolving underdispersion (less dispersion in real data than model prediction).
4. The likelihood function for model estimation depends not only on the distribution type of asymptotes, but the definition of the growth curve set as well. Consideration of these factors may be necessary, even if environmental explanatory variables and random effects are introduced.

## Graphical abstract

A growth curve set with an orderly variation is called "polymorphic" growth curve set. It is also recognized that the polymorphic growth curve set cannot be estimated using nonchronological data. However, the growth curve set can be estimated with nonchronological data using a simple maximum likelihood estimation by modeling the probability to observe a given nonchronological data.

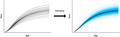

## 1 INTRODUCTION

Growth in mass is terminated when anabolism and catabolism are balanced. Thus, a finite maximal size may be generally reasonable for various quantities (e.g., length and weight) of most living beings. Therefore, most growth models feature asymptotes (Moriguchi, 2018; Richards, 1959; Turner, Bradley, Kirk, & Pruitt, 1976). Such growth models have been successfully applied to simulate and predict the growth of various quantities of various living beings, for example, the weight of mice (Bertalanffy, 1957), the body length of fishes (Pilling, Kirkwood, & Walker, 2002; Russo et al., 2009), and the height and volume of plants (Minowa, 1983; Moriguchi, Ueki, & Inoue, 2011; Nagashima, Yamamoto, & Sweda, 1980; Sweda, 1988). The use of growth models for predicting the growth of a quantity of an individual can be extended for predicting the growth of the average quantity of a group of individuals (Hagihara, 2014; Tahar et al., 2012; Trip, Clements, Raubenheimer, & Choat, 2014). In this study, the term “individual” is used to identify the subject that corresponds to one growth curve.

When we collect growth curves of many individuals, variation in the curves may be identified (Madsen & Shine, 2000; Richner, Schneiter, & Stirnimann, 1989). Variation can often be orderly, as small individuals often exhibit similar curves, but the curves differ from those of large individuals, and the curves gradually vary from small to large individuals. Richner et al. (1989) reported three types of changes in growth curves in terms of the body weight or body length of carrion crows (*Corvus corone*) due to depression:

- Decrease of the asymptote
- Decrease of the “growth constant”^1^ of the logistic model
- Decrease of both the asymptote and the growth constant

**FIGURE 1:**
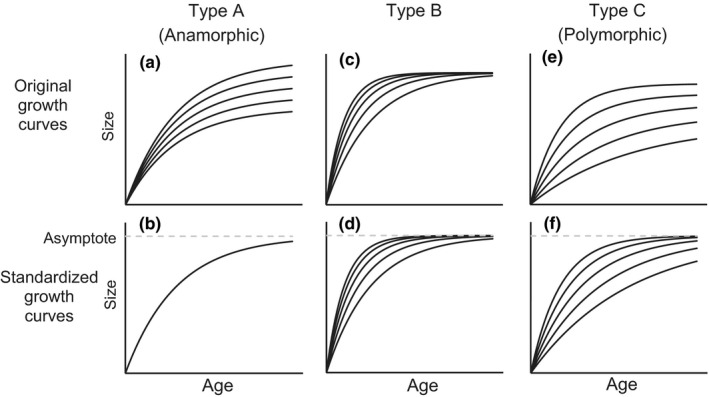
Three types of growth curve sets with orderly variations. Upper plots present the original growth curves, and lower plots present the growth curves standardized with each asymptote. After standardizing with each asymptote, the curves of Type A are identical (b). This indicates that the variation of the original growth curves in Type A (a) is caused by scaling an identical curve with various asymptotes. Type B assumes that the variation of original growth curves is caused only by the variation of the parameters of the growth curves, except for the asymptote (e.g., *k* and *b* that appear in Equation (2)). In other words, the asymptote is assumed to be identical. As a result, no differences in the shape of the curves in plots c and d exist. Type C assumes variations of both asymptotes and other parameters, with a correlation between the asymptote and the other parameters. Type C includes Type A and Type B as special cases; however, Type C does not include the growth curve sets that consist of a random mixture of various curves. Type A is termed the anamorphic growth curve set and Type C is termed the polymorphic growth curve set in this study

Note that Type C implies correlation between the asymptote and the growth constant. As a result, each type implicates that we may observe the orderly variation in the growth curves, as shown in Figure 1, with a variation in the degree of depression. Madsen and Shine (2000) presented the long‐term growth curves of the body length of water pythons (*Liasis fuscus*) to be similar to Type C, as induced by reducing the growth rate due to the “silver spoon” effect. Trip et al. (2014) suggested that, due to the temperature‐size rule, the average growth curves of two herbivorous fishes (*Odax pullus* and *Notolabrus fucicola*) in areas of high temperature, compared to those in areas of low temperature, featured a lower asymptote but faster maturity in body size. This finding suggests that Type C is reasonable for this case. The variation in growth of the stand volume is also known to exhibit Type C variation (Nishizono, Tanaka, Hosoda, Awaya, & Oishi, 2008). Similarly, growth curves of the mean tree height of numerous sites also demonstrate orderly variation of Type A–C (Burkhart & Tomé, 2012; Clutter, Fortson, Pienaar, Brister, & Bailey, 1983; Ercanli, Kahriman, & Yavuz, 2014; Scolforo et al., 2016). In this context, growth curve sets of Type A is termed the anamorphic growth curve set, as the variation of the growth curves is caused by scaling the identical curve with various asymptotes (Figure 1). In contrast, Types B and C can be termed the polymorphic growth curve set (Bailey & Clutter, 1974; Ker & Bowling, 1991). Particularly, we use the term polymorphic growth curve set to refer to Type C in this study.

**FIGURE 2:**
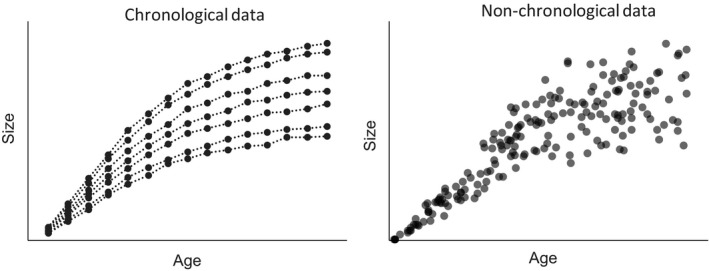
Chronological data and nonchronological data. Chronological data include the historical growth data of each individual. Nonchronological data comprise a collection of one‐time measurements of the size and age of many individuals without historical information

To estimate the growth curve set of the individuals, at least two situations emerge: We have chronological data of each individual that include the historical data series of age and size, or we have nonchronological data that do not include such historical data and only include one‐time observations of many individuals (Figure 2). If we have chronological data, and we fit a growth curve for each individual, we may simply minimize the total squared error between the value of the actual size of each measured age and the estimated age (least‐squares error estimation: LSE) (e.g., Kühleitner, Brunner, Nowak, Renner‐Martin, & Scheicher, 2019; Moriguchi, 2018; Turner et al., 1976). Similarly, with the chronological data on multiple individuals, we can estimate polymorphic models that minimize the total squared error of all the individuals or similar fitting criteria (Ercanli et al., 2014; Nunes, Patrício, Tomé, & Tomé, 2011; Tahar et al., 2012).

It is often difficult to collect sufficient chronological data due to the restricted numbers of repeatedly observable individuals (e.g., rare species) and insufficiently recorded chronological data. Even in such cases, we can often identify the age of a given individual, for example, by counting the number of tree rings or the number of rings in the otolith of fish. As a result, we can often collect the nonchronological data of many individuals, and we must estimate the growth curve set using the nonchronological data (Edminster, Mathiasen, & Olsen, 1991; Mitsuda, 2014; Mitsuda & Kitahara, 2015; Nishizono, Kitahara, Iehara, & Mitsuda, 2014).

Even with nonchronological data, we can estimate anamorphic growth curve sets using the so‐called guide curve method, which identifies the average growth curve with general nonlinear regression methods (Burkhart & Tomé, 2012; Clutter et al., 1983). In contrast, it is also recognized that polymorphic growth curve sets cannot be estimated using nonchronological data (Burkhart & Tomé, 2012; Clutter et al., 1983). Nevertheless, several researchers have attempted to estimate polymorphic growth curve sets using nonchronological data. Edminster et al. (1991) proposed a method that first applies the guide curve method and then prepares nonaverage growth curves using the 95% range of the real data variance at each age. This procedure, however, is not statistically consistent because the estimation of the average growth curve is conducted under the assumption of an anamorphic growth curve set. Furthermore, nonaverage growth curves are determined using a rough estimate without the assumption of a model of growth curve set. Socha and Tymińska‐Czabańska (2019) proposed a method that evokes chronological data with the assumption that a series of percentile values of each age in numerous nonchronological data can be assumed to be the chronological data of virtual individuals. However, the chronological data of the virtual individuals reveal quite jagged shapes that do not resemble actual growth curves, even using 5,105 data. Mitsuda (2014) attempted to estimate a polymorphic growth curve set with a hierarchical Bayesian model using WinBUGS. The Markov chain Monte Carlo sequence for the estimation was ultimately reported to not converge to a steady distribution, even with a sufficient burn‐in period. Generally, these methods attempt to overcome limitations by not modeling the process of observing nonchronological data in a straightforward manner.

**FIGURE 3:**
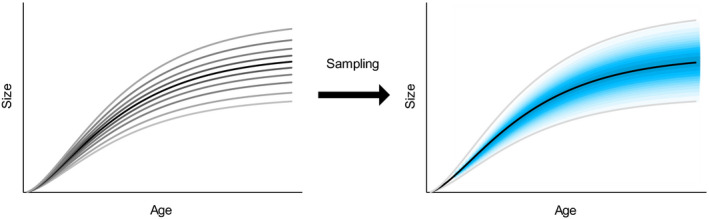
The relationship between a growth curve set and observation probability of nonchronological data. The variation of the growth curves determines the probability of observing nonchronological data

Although the purpose of this study is also to present a method to estimate a polymorphic growth curve set using nonchronological data, we aim to establish a statistically consistent method by modeling the process of obtaining a nonchronological data set in a straightforward manner. The collection process of a nonchronological data set involves random sampling from numerous growth curves. Thus, the variation of the growth curves is the fundamental cause of the variation in the collected nonchronological data sets (Figure 3). Therefore, the variation of the nonchronological data must contain information regarding the original growth curve set, and the estimation of the original growth curve set may be possible by modeling the collection process of the nonchronological data. The proposed method is developed as a simple maximum likelihood estimation (MLE) approach.

The remainder of this study is structured as follows: In Section 2.1, the anamorphic and polymorphic growth curve sets are formulated. In Section 2.2, the method and calculation technique are developed. In Section 2.3, the estimation design for the real data set of tree height is explained. In Section 2.4, the examination design of estimation ability using models with known parameters is presented. The results are reported in Section 3. Finally, in Section 4, the implications of the method on the estimation of growth curve sets are provided.

## 2 MATERIAL AND METHODS

### 2.1 Formulation of growth curve sets

First, the anamorphic and polymorphic growth curve sets were formulated.

#### 2.1.1 Anamorphic growth curve set

As shown in Figure 1, the anamorphic growth curve set has an identical curve when standardized with the asymptotes. That is, the anamorphic growth curve set assumes that the variation of the growth curves is caused by scaling an identical curve vertically with various asymptotes. As a result, the size of individual *i* at age *t_i_* can be expressed as follows:

(1) $$x_{i}=A_{i}\cdot f\left(t_{i};\theta \right)$$

where *x_i_* is the size of the individual at *t_i_*, *A_i_* is the asymptote of the individual, *f* (･) is the function with values that are in the range of [0, 1], and ***θ*** is the parameter set that is common for all individuals.

For example, Richards's (1959) function is formulated as follows:

(2) $$x=A\cdot {\left[1-\exp\left(-kt\right)\right]}^{b}$$

where *k* and *b* are the parameters that govern the standard shape of the growth curve. If *k* and *b* are common for all individuals, ***θ*** is defined as {*k*, *b*}. This formula has the structure of Equation (1).

With a given ***θ***, the asymptote of individual *i* (*A_i_*) can be estimated using *x_i_* and *t_i_* as *A_i_* = *x_i_*/*f*(*t_i_*; ***θ***). Using the estimated asymptote, the growth curve of the individual can be formulated as *x* = *A_i_* ･ *f*(*t*; ***θ***). Thus, each observation of the size and age has a growth curve.

#### 2.1.2 Polymorphic growth curve set

The polymorphic growth curve set (Type C in Figure 1) allows variation in *k* and/or *b* of Equation (2). If the variation is independent of the asymptote, we may observe a random mixture of various curves. This growth curve set is not a polymorphic growth curve set. If those parameters are correlated with an asymptote, we may find an orderly variation in growth curves. Example correlations are *b* = *b*_1_ + *b*_2_ ･ ln *A* (Cieszewski, 2004) and *k* = *k*_1_ ･ $A^{k_{2}}$ (Ker & Bowling, 1991; Mitsuda, 2014), where *b*_1_, *b*_2_, *k*_1_ and *k*_2_ are parameters. As a result, the structure of the growth curve set is as follows:

(3) $$x_{i}=A_{i}\cdot f\left(t_{i};A_{i},\theta \right)$$

Because *f* (･) includes *A_i_*, the growth curve set includes various shapes of growth curves, even after their standardization with their respective asymptotes (i.e., polymorphic). Evidently, Equation (3) includes the anamorphic growth curve set (Equation (1)) as a special case. Contrarily, Equation (3) does not include Type B in Figure 1. However, Type B can be approximated with Equation (3) in practice by assuming a very narrow distribution of the asymptotes and a large coefficient on *A_i_* in *f* (･).

### 2.2 Estimation method

We assume the following for the development of the method:

1. Asymptotes distribute according to any continuous probability distribution
2. A given growth curve set can be formulated with Equation (3)
3. Nonchronological data were sampled randomly from the growth curve set.

The first and second assumptions suggest that, strictly speaking, Type B of Figure 1 cannot be treated using this developed method. The second assumption involves that, if the asymptotes do not correlate with other parameters (e.g., *k* and *b* in Equation (2)), which suggests that the growth curves after standardization with asymptotes comprise a random mixture rather than orderly variation, then the proposed method is deemed inappropriate for the situation and random effects should instead be introduced (e.g., Paine et al., 2012). The third assumption implies the data has been sampled without sampling bias. In this case, we can consider the likelihood to obtain a given nonchronological data by formulating simple joint probability to observe the data set. The log‐likelihood (LL) function to observe a series of sizes and ages of individuals can be formulated as follows:

(4) $$\text{LL}=\ln\prod_{i}p\left(x_{i},t_{i}\right)=\sum_{i}\ln p\left(x_{i},t_{i}\right)=\sum_{i}\ln\left[p\left(t_{i}\right)p\left(x_{i}|t_{i}\right)\right]$$

where *p*(*x_i_*, *t_i_*) is the probability of observing individual *i* whose size is *x_i_* and age is *t_i_*, *p*(*t_i_*) is the probability of observing age *t_i_*, and *p*(*x_i_*| *t_i_*) is the conditional probability of observing size *x_i_* at age *t_i_*.

**FIGURE 4:**
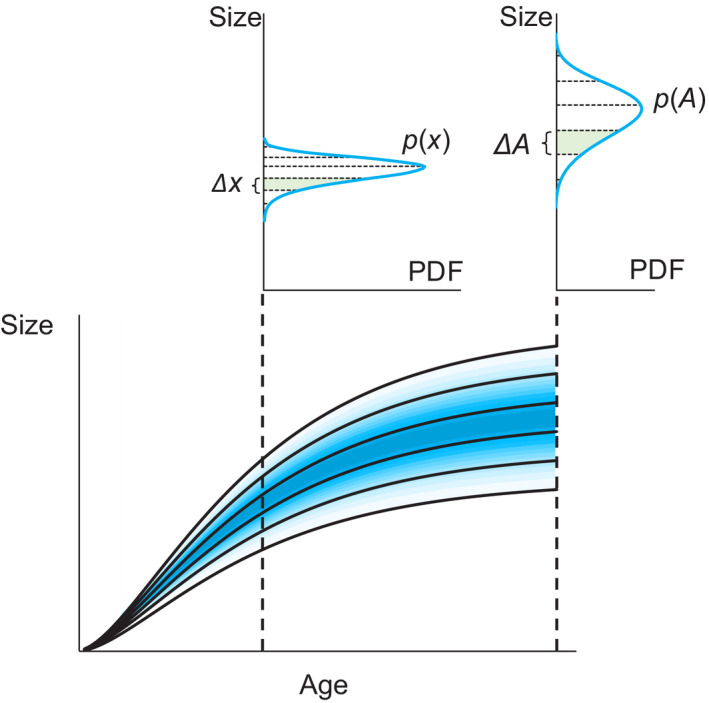
The relationship between the distributions of the asymptotes and the individual sizes at a given age. The dotted lines in the PDF plots denote the growth curves in the size‐age plot. The set of growth curves determines the relationship between the distributions, with the constraint that the areas in green must be equal to one another (Equation (5))

A set of growth curves can be a transition rule between the distributions of the asymptotes and the variation in the individual sizes at a given age (Figure 4). The transition must adhere to the following relationship:

(5) p(x)dx=p(A)dA

Note that *p*(*x*) and *p*(*A*) must not be negative. Therefore, *p*(*x*) = *p*(*A*)∙|*dA*/*dx*| = *p*(*A*)∙|*dx*/*dA*|^−1^. Using this relationship, the value of *p*(*x_i_*| *t_i_*) can be calculated as follows:

(6) $$p\left(x_{i}|t_{i}\right)=p\left(A_{i}\right)\cdot {\left(\left|{\left.\frac{\mathrm{dx}}{\mathrm{dA}}\right|}_{t_{i},A_{i}}\right|\right)}^{-1}$$

where ${\left.dx/dA\right|}_{t_{i},A_{i}}$ is the value of the derivative of the growth model with respect to an asymptote with *t_i_* and *A_i_*. As a result, the LL value can be calculated as follows:

(7) $$\begin{array}{cc} \text{LL} & =\sum_{i}\ln\left[p\left(t_{i}\right)p\left(x_{i}|t_{i}\right)\right]=\sum_{i}\left[\ln p\left(t_{i}\right)+\ln p\left(x_{i}|t_{i}\right)\right]\\ & =\sum_{i}\ln p\left(t_{i}\right)+\sum_{i}\ln p\left(x_{i}|t_{i}\right)\\ & =\sum_{i}\ln p\left(t_{i}\right)+\sum_{i}\ln p\left(A_{i}\right)+\sum_{i}\ln\left[{\left(\left|{\left.\frac{\mathrm{dx}}{\mathrm{dA}}\right|}_{t_{i},A_{i}}\right|\right)}^{-1}\right]\\ & =\sum_{i}\ln p\left(t_{i}\right)+\sum_{i}\ln p\left(A_{i}\right)-\sum_{i}\ln\left|{\left.\frac{\mathrm{dx}}{\mathrm{dA}}\right|}_{t_{i},A_{i}}\right| \end{array}$$

The first summation in the right side of Equation (7) is constant, as it is determined with only the variation of *t* in the data. Therefore, the summation can be neglected from the model estimation. The LL value will be maximized by optimizing the second and third summations.

Equation (7) implies that the guide curve method using simple weighted nonlinear regression is reasonable for the anamorphic growth curve set, and when the lognormal distribution can be assumed as the distribution of asymptotes, nonweighted LSE after logarithmic transformation is also reasonable. The statistical consistency in the use of these general methods is the reason that the anamorphic growth curve set can be estimated using nonchronological data (Burkhart & Tomé, 2012; Clutter et al., 1983). For more detail, see Appendix 1.

#### 2.2.1 Calculation procedure

To estimate the parameters of the polymorphic growth curve sets, Equation (7) must be used directly. The first summation is neglected, as it is a constant. The second summation of the right side of Equation (7) is the LL term of the asymptotes with respect to their distribution. Note that the asymptote of individual *i* (*A_i_*) can be calculated using *x_i_*, *t_i_,* and ***θ***; and for a given ***θ***, the maximal value of Equation (7) is provided when using the maximum value of the second summation. Therefore, for a given ***θ***, the distribution of asymptotes should be estimated using the MLE.

The maximal values of the second and third summations of the right side of Equation (7) for a given ***θ*** value can be calculated as follows.

1. Calculate *A_i_* based on *x_i_*, *t_i_*, and ***θ*** for all *i*. For the anamorphic growth curve sets, *A_i_* could be calculated as *x_i_*/*f*(*t_i_*; ***θ***). For the polymorphic growth curve sets, an analytical solution may not be found. Therefore, find the solution of *A_i_* of *x_i_* – *A_i_* ･ *f*(*t_i_*; *A_i_*, ***θ***) = 0 using an efficient numerical solver, for example, Brent's (1973) method.
2. Calculate the third summation of the right side of Equation (7). The derivative may be found using computer algebra systems.
3. Estimate the distribution of the asymptotes using the MLE and let the maximal log‐likelihood value be the value of the second summation in Equation (7).

Note that we need to carefully select the optimization techniques to optimize ***θ*** because the fitting problem for the growth curve sets is a nonlinear optimization problem without convexity. Similar to other fittings on nonlinear models, the fitting may not be achieved using simple downhill optimization techniques.

### 2.3 Fitting to a real data set

We present the application of the developed method using a real data set.

#### 2.3.1 Data set

We use the data set of the tree heights of cypress (*Chamaecyparis obtusa* Sieb. & Zucc.) and larch (*Larix kaempferi* (Lamb.) Carrière) trees in the Nagano region of Japan. The data set was collected by Katakura, Yamanouchi, and Furukawa (2005) to estimate the growth curve sets of the tree heights of the two species in the region. The data set includes nonchronological sets of the mean tree heights and stand‐ages of 133 cypress tree sites and 310 larch tree sites. The age distribution in the data set of each species is generally uniform and ranges from 11–110 years old for the cypress trees and 11–118 years old for the larch trees.

They collected the topographic features and the type of soil for several sites. Although using the information as additional explanatory variables may assist in explaining the variation in the data set, the variables were not used because our purpose is rather clearly to assess the modeling ability of the developed method. Ontogeny may essentially be the integration of unobservable effects; therefore, we prefer to assume that the features are unobserved. The previous research (Katakura et al., 2005) also provided anamorphic growth curve sets for the two species without using this specific information, as similar estimates based on this principle are often conducted (Mitsuda & Kitahara, 2015; Nishizono et al., 2014).

#### 2.3.2 Instance models

Although any polymorphic growth curve set that is differentiable with respect to the asymptote can be assumed, we have not tested numerous candidates given the purpose of this study. Instead, we present an application using four models, namely the combination of the two growth curve sets and two distribution types for the asymptotes.

The growth curve sets to be tested are as follows:

(8) $$x_{i}=A_{i}\cdot {\left[1-\exp\left(-kt_{i}\right)\right]}^{b}$$

(9) $$x_{i}=A_{i}\cdot {\left[1-\exp\left(-kA_{i}^{l}t_{i}\right)\right]}^{b}$$

Equations (8) and (9) with respect to the asymptotes can be derived as follows:

(10) $$\frac{\mathrm{dx}}{\mathrm{dA}}={\left[1-\exp\left(-kt\right)\right]}^{b}$$

(11) $$\frac{\mathrm{dx}}{\mathrm{dA}}=A^{l}klbtE{\left(1-E\right)}^{b-1}+{\left(1-E\right)}^{b}$$

where *E* = exp(–*kA^l^t*). Equation (8) is a major anamorphic growth curve set based on Richards's (1959) function (e.g., Clutter et al., 1983). Equation (9) is a polymorphic growth curve set that is occasionally assumed (Ker & Bowling, 1991; Mitsuda, 2014). The polymorphic growth curve set includes the anamorphic growth curve set as a case of *l* = 0.

One type of distribution of asymptotes is the lognormal distribution. Another is the generalized gamma distribution (Stacy, 1962), and its probability density function is defined as follows:

(12) $$\text{PDF}\left(x|\psi ,\tau ,\lambda \right)=\frac{\tau }{\Gamma (\psi )\cdot \lambda }{\left(\frac{x}{\lambda }\right)}^{\psi \tau -1}\exp\left[-{\left(\frac{x}{\lambda }\right)}^{\tau }\right]$$

where Γ(∙) is the gamma function, *λ* is the scale parameter, and both *τ* and *ψ* are shape parameters. The generalized gamma distribution includes the gamma distribution with *τ* = 1 and the Weibull distribution with *ψ* = 1. The lognormal distribution is approximated using *ψ* → ∞ (Prentice, 1974).

With the combination of the growth curve sets and the distributions of the asymptotes, we define four models: anamorphic‐lognormal (AL), anamorphic‐generalized‐gamma (AG), polymorphic‐lognormal (PL), and polymorphic‐generalized‐gamma (PG).

The lower and upper bounds in the search for the optimal value of each parameter are determined experimentally with repeated trials, thereby allowing the optimal parameter set to be included. For AL and AG, the ranges of *k* and *b* are [10^−12^, 0.1] and [0.01, 5], respectively. For PL and PG, *A^l^* can be a very large value with a large *l*. This causes difficulty in determining the candidate range of *k*. To avoid this issue, we replace Equation (5) with the following formula at optimization:

(13) $$x_{i}=A_{i}{\left\{1-\exp\left[-k^{\prime }{\left(A_{i}/25\right)}^{l}t_{i}\right]\right\}}^{b}$$

where 25 is the roughly estimated average value of the asymptotes. The ranges of *k*′, *b*, and *l* are [10^−12^, 0.1], [0.01, 5], and [–10, 10], respectively. We then calculate the value of *k* as *k*′/25*^l^*.

#### 2.3.3 MLE calculation

To detect the parameter set that maximizes the log‐likelihood value, we use the particle swarm optimization technique (PSO; Kennedy and Eberhart, 1995), which is an efficient optimization algorithm for general optimization problems with continuous variables and fixed ranges. For more detail, see Appendix 2. 95% confidence intervals of the parameters are also estimated using the empirical bootstrapping method (Efron, 1979, 1981) with 1,001 resamples from the data source and application of the MLE for each resampled data set.

#### 2.3.4 Comparison

We report the AIC values (Akaike, 1973) of each model. Although the model with a minimum AIC value has the strongest support, the models with AIC values that differ from the minimum AIC value by <2 also are supported (Burnham & Anderson, 2004). When the models with AIC values differ from the minimum AIC value by discrepancies larger than 10, the models can be considered to have essentially no support (Burnham & Anderson, 2004).

We also conduct the likelihood ratio test between comparable models using the *χ* ^2^ distribution and negative log‐likelihood (NLL) values. The likelihood ratio test can be applied to compare the goodness of fit of models with a “nested” relationship (Lewis, Butler, & Gilbert, 2011; MacKenzie et al., 2018). Therefore, the likelihood ratio test cannot be conducted between the AG and PL models because of their nonnested relationship. Lewis et al. (2011) presented an approach to perform the likelihood ratio test between nonnested models for count data with a simulative calculation that employs the test criterion. However, in our case, we focus on the differences in the goodness of fit between the anamorphic and polymorphic growth curve sets and between the lognormal and generalized gamma distributions. Therefore, we use the ordinary likelihood ratio test and AIC values to conduct the model comparisons.

The numbers of model parameters are 4 for AL, 5 for AG and PL, and 6 for PG. These values are used to calculate the AIC values and the degrees of freedom for the likelihood ratio test.

### 2.4 Model reproductivity test

Whether the method has ability to reproduce “true” models is tested using the models estimated for the cypress and larch data as the “true” models. Similar to parametric bootstrapping (Genest & Rémillard, 2008), we repeatedly execute the following procedure:

1. Generate virtual data from the true models. The number of data is 100 or 200.
2. Using the virtual data, we attempt to estimate the models without information on the parameters of the true models.

These procedures are executed 1,001 times independently, and the estimated growth curves and those of the true models are compared. The estimation method in the second procedure is that explained in Section 2.3. Not only are the original models estimated with properly modeled cases (e.g., the case in which both the “true” model and the estimator are PG), we also test how the prediction of the growth curves biases when nonproper models (e.g., the case in which the “true” model is PG but the estimator is PL) are assumed. We evaluate the range of estimations of three growth curves using either the median, 2.5th, or 97.5th percentile asymptotes.

## 3 RESULTS

### 3.1 Application with real data set

**FIGURE 5:**
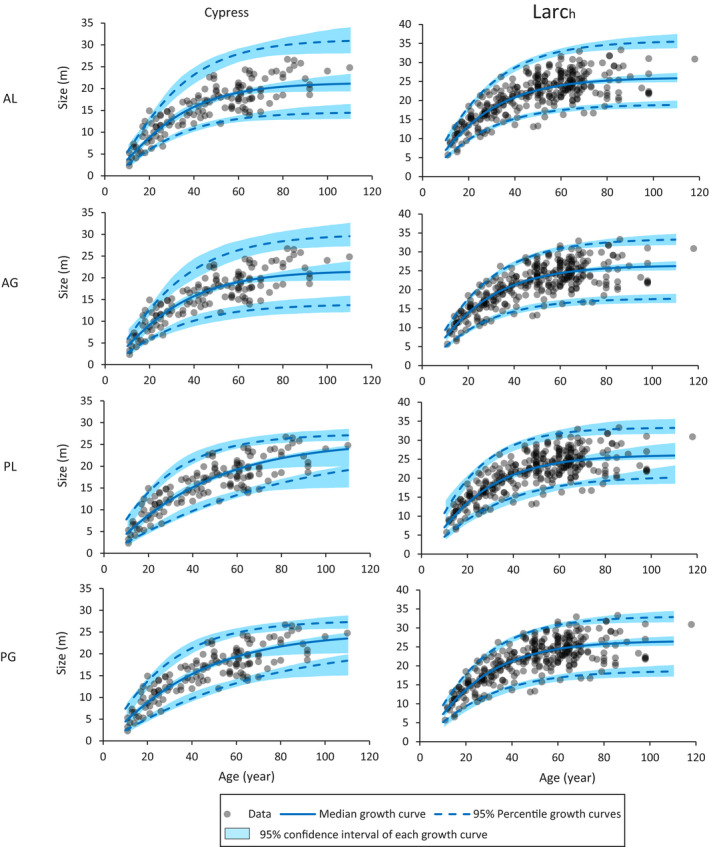
Original data and growth curves estimated with MLE. Solid lines indicate the growth curves with median asymptotes. Dotted lines indicate the growth curves with 2.5th and 97.5th percentile asymptotes. Shadows indicate the 95% confidence interval of each curve

The *χ* ^2^ statistics of the likelihood ratio tests are reported in Table 1. The estimated parameters and fitting criteria are shown in Table 2. The relationship of the original data, and the growth curves with the median, 2.5th, and 97.5th percentile asymptotes of the estimated models are shown in Figure 5.

**TABLE 1:** *χ* ^2^ statistics of the likelihood ratio tests

Model	Cypress				Larch			
	AL	AG	PL	PG	AL	AG	PL	PG
AL	—	—	—	—	—	—	—	—
AG	2.26	—	—	—	28.80^***^	—	—	—
PL	18.78^***^	—^a^	—	—	7.65^**^	—^a^	—	—
PG	19.13^***^	16.87^***^	0.35	—	30.77^***^	1.97	23.12^***^	—

Note

Significance level: **: 1%, ***: 0.1%. No symbol indicates no significant difference at the 10% level. Degree of freedom: 1 for tests between AL‐AG, AL‐PL, AG‐PG, and PL‐PG. 2 for tests between AL‐PG.

^a^ Test was not conducted because of nonnested relationship.

**TABLE 2:** Estimated parameters and fitting criteria

Species	Model	Growth curve set			Lognormal		Generalized gamma			Fitting criteria	
		*k*	*b*	*l*	*μ*	*σ*	*λ*	*ψ*	*τ*	NLL	AIC
Cypress	AL	0.0463 [0.0234, 0.0731]	1.76 [1.04, 2.86]	—	3.06 [2.96, 3.23]	0.194 [0.166, 0.216]	—	—	—	327.0	662.0
	AG	0.0413 [0.0172, 0.0720]	1.50 [0.81, 2.83]	—	—	—	11.9 [0.00, 28.0]	4.73 [0.546, 3,250.1]	2.47 [0.10, 9.94]	325.9	661.7
	PL	0.0190 [0.0062, 0.0689]	1.16 [0.92, 2.34]	8.29 [0.43, 10.00]	3.25 [3.00, 3.33]	0.031 [0.023, 0.146]	—	—	—	317.6	**645.2**
	PG	0.0278 [0.0062, 0.0671]	1.22 [0.92, 2.30]	3.85 [0.43, 10.00]	—	—	8.70 [0.00, 24.2]	30.98 [2.08, 1.59 ･ 10^5^]	3.25 [0.10, 10.0]	317.4	646.8
Larch	AL	0.0471 [0.0332, 0.0614]	1.35 [0.98, 1.80]	—	3.26 [3.21, 3.33]	0.161 [0.146, 0.175]	—	‐	—	810.1	1,628.3
	AG	0.0467 [0.0344, 0.0613]	1.29 [0.98,1.72]	—	—	—	24.9 [16.4, 28.5]	1.70 [0.72, 4.86]	5.48 [3.24, 9.96]	795.7	**1,601.5**
	PL	0.0488 [0.0016, 0.0609]	1.32 [0.72, 1.80]	0.75 [−0.10, 10.00]	3.26 [3.22, 3.48]	0.123 [0.029, 0.164]	—	—	—	806.3	1,622.6
	PG	0.0452 [0.0317, 0.0600]	1.30 [0.98, 1.75]	0.27 [−0.15, 0.80]	—	—	24.1 [15.7, 29.6]	2.03 [0.82, 5.56]	5.48 [3.24, 9.97]	794.8	**1,601.5**

Note

The values in the brackets are the 95% confidence intervals of each parameter that were determined with the empirical bootstrap method.

For the cypress data, the likelihood ratio tests between AL and PL and between AG and PG indicate that the latter models have better fittings at a 0.1% significance level, respectively (Table 1). Contrarily, between AL and AG and between PL and PG, a significant difference in goodness‐of‐fit was not suggested using the likelihood ratio test at the 10% level. As shown in Table 2, the AIC values of PL and PG were much smaller than those of AL and AG, albeit the AIC values are similar for AL and AG and for PL and PG. The AIC value of the PL model is the smallest of the four. The PG model can also be supported, because the difference of its AIC value compared with that of the PL model was <2. The 95% confidence intervals of *l* for both PL and PG do not include zero. The upper values of the 95% confidence intervals of *ψ* for both AG and PG are large, that is, they tend to approximate lognormal distributions. In Figure 5, both growth curves of AL and AG for the cypress data with the 2.5th and 97.5th percentile asymptotes are underestimated and overestimated, respectively, compared with the original data distribution, especially for older age. Thus, the models with the anamorphic growth curve set involve underdispersions, that is, the data set exhibits less dispersion compared to the model prediction. This underdispersion was not found for the PL and PG models, which assume the polymorphic growth curve set.

For the larch data, the likelihood ratio test suggested that the PL model has a better fit compared to the AL model at the 1% significant level (Table 1). However, no significant difference was found between PG and AG at the 10% level. When comparing the AL and AG models and subsequently the PL and PG models, the latter models have better fits at the 0.1% significance level. Because the NLL of the PG model is slightly smaller than that of AG, the AIC values of the PG and AG models are the same, although much smaller than those of AL and PL (Table 2). The 95% confidence intervals of *l* for both the PL and PG models include zero, while the upper values of the 95% confidence intervals of *ψ* for both the AG and PG models are much lower than the respective cases with the cypress trees. In Figure 5, all estimated growth curves of the AG and PG models seem to be approximately equivalent.

### 3.2 Model reproductivity

**FIGURE 6:**
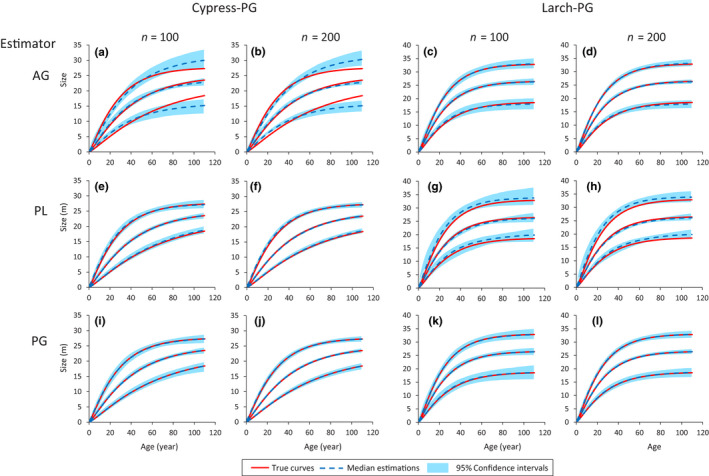
Estimation of growth curves using virtual data generated by PG models for cypress and larch. *n* indicates the number of data per estimation. Red lines indicate the growth curves of the original models with 2.5th, median and 97.5th percentile asymptotes. Blue shadows indicate the 95% confidence interval of each curve. Blue dotted lines are the median estimation for each curve

Because the PG model provided good fitting results for the two species, the MLE‐estimated PG models of the cypress and larch were used as the “true” models (Cypress‐PG and Larch‐PG). The models were estimated assuming AG, PL, and PG as the estimator. The results are shown in Figure 6.

When using the PG model (i.e., proper model) as the estimator, the median estimation of each growth curve overlaps the curves of the true models (Plots i‐l in Figure 6). This is common for both *n* (number of data) = 100 and *n* = 200 cases, whereas the cases of *n* = 200 demonstrate narrower 95% confidence intervals for each growth curve. Similar results can be found for the case in which the PL model was used as the estimator for the Cypress‐PG (Plots e,f in Figure 6) model. This is natural, as the distribution of the asymptotes of the Cypress‐PG model approximates a lognormal distribution; consequently, the PL model can approximate the PG model for this case. Similarly, the growth curves of the Larch‐PG model could be reproduced with small bias using AG as the estimator (Plots c,d in Figure 6), given the similar AIC values in the previous analysis.

In contrast, when estimating the Cypress‐PG model using the AG model as the estimator, the confidence intervals of the estimations for the curves with the 2.5th and 97.5th percentile asymptotes failed to include the curves of the true model (Plots a,b in Figure 6). In this case, an increase in the number of data (*n* = 100 to *n* = 200) did not improve the bias. Estimation of the Larch‐PG model using PL as the estimator also demonstrated biases (Plots g,h in Figure 6); the median estimations for the growth curves with the 2.5th and 97.5th percentile asymptotes tended to be overestimated, whereas the median estimation for the curves with the median asymptote tended to be underestimated.

## 4 DISCUSSION

In the application process using real data, the AL model was found to be not supported for both species. The nonweighted LSE value of log‐transformed size can be used to estimate the AL model (Appendix 1); however, this finding indicates that the LSE method is not effective for the data of both species. For the larch data, the AG model and the PG model exhibited the lowest AIC values; but the PG model demonstrated negligible differences in estimating the growth curve sets compared to the AG model (Figure 5). This indicates that relying on the assumption of the anamorphic growth curve set for the larch data is reasonable. Contrarily, for the cypress data, the AIC values of the PL and PG models were much smaller than those of the AL and AG models. In fact, compared to the estimated growth curves using the 2.5th and 97.5th percentile asymptotes for AL and AG, real data suggest less variation than expected based on the estimated model at old age (Figure 5), that is, the models displaying underdispersion. Reasonable variation in size at old age must be observed if the models are deemed correct; therefore, the models are not supported. Contrarily, the growth curves of the PL and PG models considerably explain the variation in size at old age. As a result, the use of the polymorphic growth curve sets was strongly supported for the cypress data.

In the reproduction test of the “true” models, the overlap of median estimations on the growth curves of the true (PG) models using the proper (PG) model or compatible models (PL for the cypress data and AG for the larch data) indicates that the proposed method demonstrates reasonable estimation ability. Naturally, the method provides biased estimation when suitable models are not used (i.e., AG for cypress and PL for larch). The biases are not improved with an increase in the number of samples (Plots a,b in Figure 6). In practice, we usually do not know the “true” model of the growth curve sets. The proposed method provides a way to compare various models to identify a relatively appropriate model for given nonchronological data. The appropriate model should be identified through the comparison between candidate models. Therefore, even with the proposed method, preparation of many models with possible distribution types for asymptotes and growth curve sets and selection of the best models using the fitting criteria are necessary steps.

The assumptions of the proposed method presented in Section 2.2 are similar to those of the estimation techniques proposed by Edminster et al. (1991) and Socha and Tymińska‐Czabańska (2019). Edminster et al. (1991) first applied the guide curve method, which can essentially be applied for only the anamorphic growth curve set, and then nonaverage growth curves were roughly estimated given the variation of the real data of each age class. Socha and Tymińska‐Czabańska (2019) assumed that a series of percentile values of each age in a given data set can be treated as virtual chronological data of virtual individuals. With the virtual chronological data, the estimation method for chronological data was applied. These two methods assume that the growth curve sets fundamentally determine the variation in the data set, similar to our method. These methods, however, introduced a special assumption, which is that the average growth curve can be estimated using an ordinary method for the anamorphic growth curve set, or the series of percentile values of all ages in a given data set can be treated as virtual chronological data to use an ordinary fitting method.

Mitsuda (2014) attempted to prepare a polymorphic growth curve set with a hierarchical Bayesian model using nonchronological data, but reported that the Markov chain Monte Carlo sequence for the estimation did not converge. This attempt did not take into account that both the distribution of the asymptotes and the growth curve set determines the variation of individual sizes at a given age (Equation (6)). We instead developed a method that is statistically straightforward by modeling the sampling process of nonchronological data.

For the polymorphic growth curve sets, Equation (6) does not simply scale the distribution of the asymptotes, but also changes the shapes of the distribution of the sizes at a given age. This may lead to a potential issue: What is the essential distribution of the individual sizes? In other words, why do we assume that the asymptotes obey well‐known distributions? In fact, the method we developed can use any distribution function as the asymptotes, because it was developed without specification of the function. Similarly, the distribution of the sizes at a given standard (finite) age can be used, instead of the distribution of asymptotes, to develop the method with a few modifications (Appendix 3). However, it may be impossible to identify such a “fundamental” distribution type for a “fundamental” age. This is the reason that we assumed that the distribution of asymptotes obeys a well‐known distribution in the application: there is no reason to assume a special distribution and standard ages.

For simplicity, we estimated the growth curve sets of the data sets without topographical variables, as ontogeny integrates the effects. However, it is possible that environmental parameters explain the differences in the parameters of the growth curve sets (***θ***) and the distribution of the asymptotes (Mitsuda, 2014; Nishizono et al., 2014; Nunes et al., 2011). The LL value proposed in Equation (7) may be simply extended by adding the dependencies of ***θ*** and the distribution parameters of the asymptotes on the explanatory variables. Estimation methods, including random effects, may also be developed by customizing the LL value in Equation (7). Our study indicates that, in both cases, the estimation of the polymorphic growth curve sets requires the use of the LL value that takes into account that a growth curve set regulates the observed data distribution.

In this paper, we did not consider the mortality of individuals. Although this may not serve as a bias in our application case, given that we used mean tree height data of a particular region, this may serve as potential bias when being applied to the data set of individuals with nonnegligible mortality rates. Even for this case, an applicable likelihood function may be developed with further extension in a future study.

## CONFLICT OF INTEREST

None declared.

## AUTHOR CONTRIBUTION

**Kai Moriguchi:** Conceptualization (equal); data curation (equal); formal analysis (equal); funding acquisition (equal); investigation (equal); methodology (equal); project administration (equal); resources (equal); software (equal); validation (equal); visualization (equal); writing‐original draft (equal); Writing – review and editing (equal).

## Data Availability

Java code set for the estimation: Dryad https://doi.org/10.5061/dryad.g4f4qrfn4.

## ENDNOTE

^1^ The coefficient of age similar to *k* of Equation (2) in this paper. The parameter governs the standard shape of the growth curves.

## APPENDIX 1 Special cases for the anamorphic growth curve sets

For the anamorphic growth curve sets, due to Equation (1), *dx*/*dA* = *f*(*t*; ***θ***). Therefore, Equation (6) can be simply derived as follows:

(A1) $$p(x_{i}|t_{i})=p\left(A_{i}\right)\cdot \frac{1}{f(t_{i};\theta )}$$

Additionally, anamorphic growth curve sets imply a constant coefficient of variation with respect to *t* (Burkhart & Tomé, 2012).^2^ It indicates that the distribution of individual sizes at a given age is prepared by simply scaling the distribution of the asymptotes with *f*(*t*; ***θ***). Therefore, Equation (7) includes the fitting criterion of simple nonlinear regression weighting data with 1/*f*(*t*; ***θ***) for the anamorphic growth curve sets.

The nonweighted LSE after the log‐transform of the individual sizes is also included when assuming a lognormal distribution as the distribution type of the asymptotes. In this case, the second summation of the right side of Equation (7) is derived as follows:

(A2) $$\begin{array}{cc} \sum_{i}\ln p\left(A_{i}\right) & =\sum_{i}\ln\left\{\frac{1}{\sqrt{2\pi \sigma^{2}}}\exp\left[-\frac{{\left(\ln A_{i}-\mu \right)}^{2}}{2\sigma^{2}}\right]\frac{1}{A_{i}}\right\}\\ & =\sum_{i}\ln\frac{1}{\sqrt{2\pi \sigma^{2}}}-\frac{1}{2\sigma^{2}}\sum_{i}{\left(\ln A_{i}-\mu \right)}^{2}+\sum_{i}\ln\frac{1}{A_{i}}\\ & =-\frac{n}{2}\ln\left(2\pi \sigma^{2}\right)-\frac{1}{2\sigma^{2}}\sum_{i}{\left(\ln A_{i}-\mu \right)}^{2}-\sum_{i}\ln A_{i} \end{array}$$

where *μ* and *σ* are the location and scale parameters, respectively. For the anamorphic growth curve sets, the logarithmic values of the individual sizes are as follows (see Equation (1)):

(A3) $$\ln x_{i}=\ln A_{i}+\ln f\left(t_{i};\theta \right)$$

Using Equation (A3), the third term of the right side of Equation (A2) can be formulated as follows:

(A4) $$-\sum_{i}\ln A_{i}=-\sum_{i}\ln x_{i}+\sum_{i}\ln f\left(t_{i};\theta \right)$$

Because *dx*/*dA* = $f\left(t;\theta \right)$ for the anamorphic growth curve set, the third summation of Equation (7) is calculated as follows:

(A5) $$-\sum_{i}\ln\left({\left.\frac{\mathrm{dx}}{\mathrm{dA}}\right|}_{t_{i},A_{i}}\right)=-\sum_{i}\ln f\left(t_{i};\theta \right)$$

Using Equations (A2), (A4), and (A5), the second and third summations of the right side of Equation (7) are as follows:

(A6) $$\begin{array}{cc} \sum_{i}\ln p\left(A_{i}\right)-\sum_{i}\ln\left({\left.\frac{\mathrm{dx}}{\mathrm{dA}}\right|}_{t_{i},A_{i}}\right)\\ & =-\frac{n}{2}\ln\left(2\pi \sigma^{2}\right)-\frac{1}{2\sigma^{2}}\sum_{i}{\left(\ln A_{i}-\mu \right)}^{2}-\sum_{i}\ln x_{i}+\sum_{i}\ln f\left(t_{i};\theta \right)-\sum_{i}\ln f\left(t_{i};\theta \right)\\ & =-\frac{n}{2}\ln\left(2\pi \sigma^{2}\right)-\frac{1}{2\sigma^{2}}\sum_{i}{\left(\ln A_{i}-\mu \right)}^{2}-\sum_{i}\ln x_{i} \end{array}$$

Note that the third term of the right side of Equation (A6) is constant for a given dataset. The first and second terms of the right side of Equation (A6) are the LL function of the normal distribution with respect to ln *A_i_* whose parameters can be identified by minimizing the second summation of the right side. As is well known, $\sum_{i}\left(\ln A_{i}\right)/n$ and $\sqrt{\sum_{i}{\left\{\left(\ln A_{i}\right)-\mu \right\}}^{2}/n}$ are the MLE values of *μ* and *σ*, respectively.

Furthermore, let us define the “base” curve with $\exp\left(\mu \right)\cdot f\left(t_{i};\theta \right)$. Using Equation (A3), the log‐transformed estimation value ( ${\hat{x}}_{i}$) of the base curve at *t_i_* is given as follows:

(A7) $$\ln{\hat{x}}_{i}=\mu +\ln f\left(t_{i};\theta \right)$$

Eliminating $\ln f(t_{i};\theta )$ from Equations (A3) and (A7), $\ln A_{i}-\mu =\ln x_{i}-\ln{\hat{x}}_{i}$ is derived. Therefore, the following relationship is noted:

(A8) $$\sum_{i}{\left(\ln A_{i}-\mu \right)}^{2}=\sum_{i}{\left(\ln x_{i}-\ln{\hat{x}}_{i}\right)}^{2}$$

As a result, the maximization of Equation (A6) implies the minimization of the right side of Equation (A8), that is, LSE after the log‐transform of size, *x_i_*.

In this case, the LSE is derived under the assumption that the variation in size at a given age is caused by the variation in the asymptotes. Note that the LSE estimation of the growth curve of an individual using the chronological data assumes that the discrepancy between actual and estimated sizes is the estimation error. Similarly, if all individuals must follow an identical growth curve, LSE estimation for the curve using nonchronological data of many individuals is consistent. Thus, LSE can be a consistent method for these two cases; however, the assumptions on the variation in the growth curves are fundamentally different, and the cases should be distinguished.

## APPENDIX 2 Particle swarm optimization

PSO allows many particles to seek the optimum condition by simulating the dynamics of swarms through the reciprocal communication between particles. Each particle moves in the search space according to the inertia of the particle, the memory of the best position of the particle, and the information of the best position of all the particles. It is known that the PSO can provide high optimization ability for multidimensional optimization problems (Chaitou & Nika, 2012; Ishigame & Yasuda, 2008; Robinson & Rahmat‐Samii, 2004; Shi & Eberhart, 1999). The original model controls the velocity and positions of each particle as follows:

(B1) $$V_{k+1}^{m}=wV_{k}^{m}+c_{1}r_{k}^{m}\cdot \left(P_{k}^{m}-X_{k}^{m}\right)+c_{2}s_{k}^{m}\cdot \left(Q_{k}-X_{k}^{m}\right)$$

(B2) $$X_{k}^{m+1}=X_{k}^{m}+X_{k+1}^{m}$$

where *m* identifies a particle; *k* indicates the simulation time; ***V*** is the velocity vector; *r* and *s* are random numbers generated with the standard uniform distribution ([0, 1]), respectively; ***X*** is the position of the particle; ***P*** is the best position of the particle as of yet; ***Q*** is the best position of all the particles; and *w*, *c*_1_, and *c*_2_ are given parameters. In our case, the numbers of dimensions of ***V***, ***P***, ***X***, and ***Q*** are the same as the dimension of ***θ***.

Various derived techniques exist (Eberhart & Shi, 2000; Ishigame & Yasuda, 2008). This study employed Fourie and Groenwold's (2002) method, which has been reported to be an efficient method compared to alternatives (Ishigame & Yasuda, 2008; Schutte & Groenwold, 2005). The method adaptively changes *w* and the maximum velocity of each particle as follows:

(B3) $$w\leftarrow \alpha w,\;V_{\max}\leftarrow \beta V_{\max}\;\text{if}\;f\left(Q_{k}\right)\geq f\left(Q_{k-h}\right)$$

where *α*, *β*, and *h* are parameters; ***V***_max_ is the maximum velocity vector; and *f* (･) is the function to be minimized. We use NLL as this function. The initial maximum velocity is given as follows:

(B4) $$V_{\max}=\gamma \left(X_{U}-X_{L}\right)$$

where ***X*** *_U_* and ***X*** *_L_* are the vectors of the upper and lower bounds of the variables, respectively; and *γ* is a parameter.

According to Fourie and Groenwold (2002), the *γ* value and the initial *w* value are set to 0.4 and 1.4, respectively. Schutte and Groenwold (2005) recommended using *c*_1_ = *c*_2_ = 2, *h* = 10, and *α* = *β* = 0.99 as the parameters of the PSO for good optimization performance. Although Schutte and Groenwold (2005) reported that the use of 20 particles is reasonable to balance computational costs and reliability, 56 particles were used to increase reliability. The PSO stops when the number of iterations reaches 1,000 or the difference between the minimum and median values of the NLL in the particles becomes less than 10^−8^.

## APPENDIX 3 Using the distribution of sizes at a given age, rather than that of asymptotes

Instead of using a distribution of asymptotes, we can develop a method that uses a distribution of sizes at a given standard age. With a definite standard age established, let *X* be the size of individuals at the standard age. Even for this case, the following equation emerges (see explanation for Equation (5)):

(C1) p(x)dx=p(X)dX

Similar to Equation (6), the value of *p*(*x_i_*|*t_i_*) can be calculated as follows:

(C2) $$p\left(x_{i}|t_{i}\right)=p\left(X_{i}\right)\cdot \left|{\left.\frac{\mathrm{dX}}{\mathrm{dx}}\right|}_{t_{i},X_{i}}\right|=p\left(X_{i}\right)\cdot {\left(\left|{\left.\frac{\mathrm{dx}}{\mathrm{dX}}\right|}_{t_{i},X_{i}}\right|\right)}^{-1}$$

Because *dx*/*dX* = *dx*/*dA* ∙ *dA*/*dX* = *dx*/*dA* ∙ (*dX*/*dA*)^−1^, the value of *dx*/*dX* can be calculated with the formula of *dx*/*dA*. The LL value can be formulated simply by replacing *A* of Equation (7) with *X*.
